# A prospective, controlled study on the utility of rotational thromboelastometry in surgery for acute type A aortic dissection

**DOI:** 10.1038/s41598-022-23701-z

**Published:** 2022-11-08

**Authors:** Mårten Larsson, Igor Zindovic, Johan Sjögren, Peter J. Svensson, Karin Strandberg, Shahab Nozohoor

**Affiliations:** 1 Department of Clinical Sciences, Department of Cardiothoracic Surgery, Skåne University Hospital, Lund University, 221 85 Lund, Sweden; 2 Department of Coagulation Disorders, Skåne University Hospital, Lund University, Malmö, Sweden; 3 University and Regional Laboratories, Region Skåne, Malmö, Sweden

**Keywords:** Cardiovascular biology, Outcomes research

## Abstract

To evaluate the hemostatic system with ROTEM in patients undergoing surgery for acute type aortic dissection (ATAAD) using elective aortic procedures as controls. This was a prospective, controlled, observational study. The study was performed at a tertiary referral center and university hospital. Twenty-three patients with ATAAD were compared to 20 control patients undergoing elective surgery of the ascending aorta or the aortic root. ROTEM (INTEM, EXTEM, HEPTEM and FIBTEM) was tested at 6 points in time before, during and after surgery for ATAAD or elective aortic surgery. The ATAAD group had an activated coagulation coming into the surgical theatre. The two groups showed activation of both major coagulation pathways during surgery, but the ATAAD group consistently had larger deficiencies. Reversal of the coagulopathy was successful, although none of the groups reached elective baseline until postoperative day 1. ROTEM did not detect low levels of clotting factors at heparin reversal nor low levels of platelets. This study demonstrated that ATAAD is associated with a coagulopathic state. Surgery causes additional damage to the hemostatic system in ATAAD patients as well as in patients undergoing elective surgery of the ascending aorta or the aortic root. ROTEM does not adequately catch the full coagulopathy in ATAAD. A transfusion protocol in ATAAD should be specifically created to target this complex coagulopathic state and ROTEM does not negate the need for routine laboratory tests.

## Introduction

Acute type A aortic dissection (ATAAD) is a lethal condition requiring emergent aortic surgery. The procedure is challenging and associated with high rates of peri-operative bleeding and blood product transfusions. Mortality is high with rates ranging from 16 to 18% after surgery in large registries^1–3^. A significant cause of the high mortality and morbidity is peri-operative bleeding^4^. The impairment of the coagulation system is caused by the contact of blood with the non-endothelialized walls of the false lumen, but other triggers also have been suggested^5–7^. Surgery for acute type A aortic dissection is conducted using a cardiopulmonary bypass circuit and usually a deep hypothermic state, both of which further increase the coagulopathy.

Transfusion strategy in major aortic surgery may either be reactive, with the administration of blood products in response to the development of a clinical coagulopathy, or pre-emptive, based on the administration of blood products to prevent a clinically detectable coagulopathy. Both approaches lead to a significant use of blood products. A recent review of bleeding management in vascular surgery concluded that hemostasis should be monitored, and goal directed^8^. This calls for alternative bleeding management, and the adoption of a transfusion algorithm guided by rotational thromboelastometry (ROTEM) could be an option.

At many centers, management of perioperative bleeding is carried out according to ROTEM-guided protocol for transfusion of blood products and procoagulants, reducing the need for transfusions and decreasing the risk of bleeding after cardiac surgery^9–12^.

A prospective study by Ogawa et al.^13^ compared values obtained using standard laboratory coagulation tests (PT(INR), APTT and fibrinogen) with ROTEM clotting time (CT) and maximum clot firmness (MCF) values for the parameters INTEM, EXTEM, HEPTEM, and FIBTEM in adult patients undergoing cardiac surgery and demonstrated that some ROTEM measurements could act as surrogates for standard coagulation tests. However, although reference ROTEM values in patients undergoing elective and non-complex cardiac surgery have been described^14–16^, similar data are lacking in the acute and complex setting. Furthermore, few studies have investigated the dynamics of the key components of the coagulation system visualized by ROTEM in association with the complex coagulopathic setting of ATAAD surgery. It is therefore unclear whether the ROTEM-guided treatment algorithm utilized in routine surgery also may be used when performing ATAAD repair.

To assess the performance of ROTEM during surgery for ATAAD, we reviewed ROTEM in surgically treated patients with ATAAD compared to a control group undergoing elective aortic surgery.

## Methods

This study was approved by the Ethics Committee for Clinical Research at Lund University, Sweden (ref. 2015/197) in accordance with the declaration of Helsinki. As this was one of several studies derived from the same prospective project, the study population and methods have been described previously^17^.

### Study population

A total of 27 patients with ATAAD were selected for the study. Of those, four patients were excluded: two because they did not follow surgical protocol, one because of intraoperative death, and one because of a lack of resources. The control group consisted of 20 patients who underwent elective surgery of the ascending aorta and/or the aortic root. A preoperative written or oral consent by the patient or the next of kin was retrieved before any collection of data in all ATAAD patients. A preoperative written consent was obtained by all elective control patients, in patients operated for ATAAD, the informed consent was given by either the patient or the next of kin orally and/or written.

### Study design

This was a prospective, single center, observational study comparing patients undergoing surgery for ATAAD with a control group consisting of patients undergoing elective aortic surgery of the ascending aorta and/or the aortic root. The ATAAD group consisted of patients over the age of 18 with symptom duration < 48 h undergoing surgery for ATAAD at Skåne University Hospital, Lund, Sweden, between September 2015 and April 2018. ATAAD was confirmed by contrast-enhanced computed tomography. The anatomical extent of the dissection was defined according to the Stanford^18^ and Debakey^19^ classification. Exclusion criteria were preoperative use of anti-coagulants or anti-platelet drugs other than aspirin (both groups) and if surgical approach deviated from routine (as described below) (ATAAD group).

**Figure 1:**
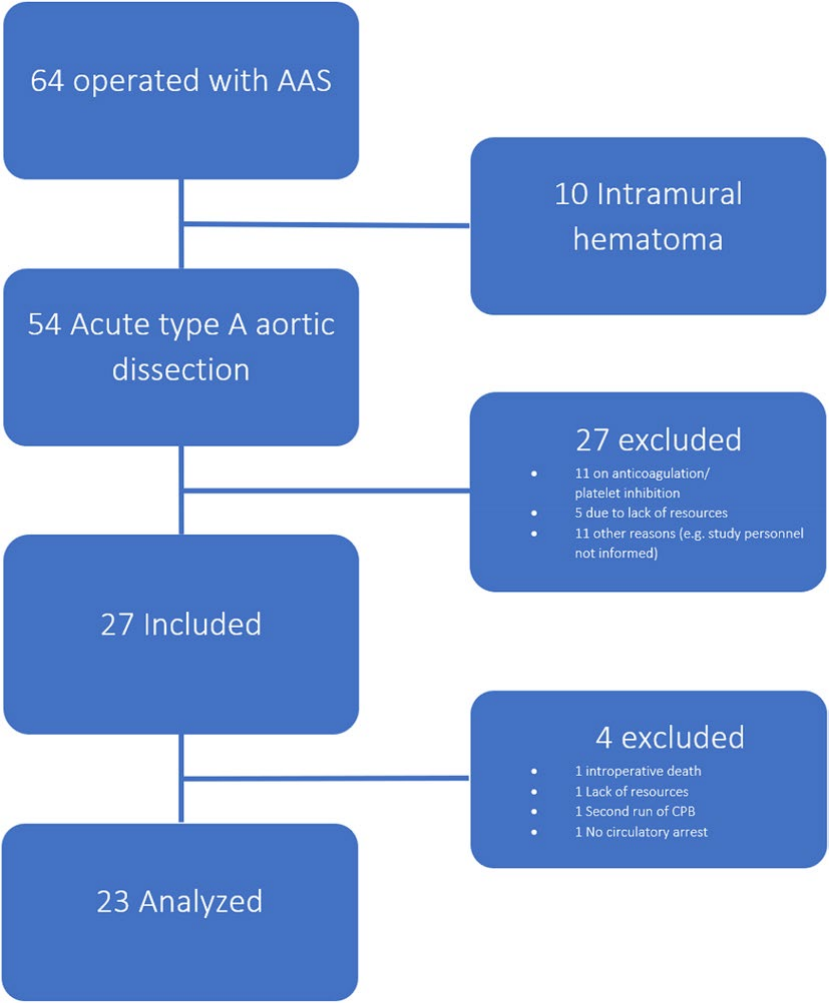
Flow chart of patients with acute aortic syndromes referred to the Department of Cardiothoracic Surgery, Skåne University Hospital, Lund, Sweden, during the inclusion period.

**Figure 2:**
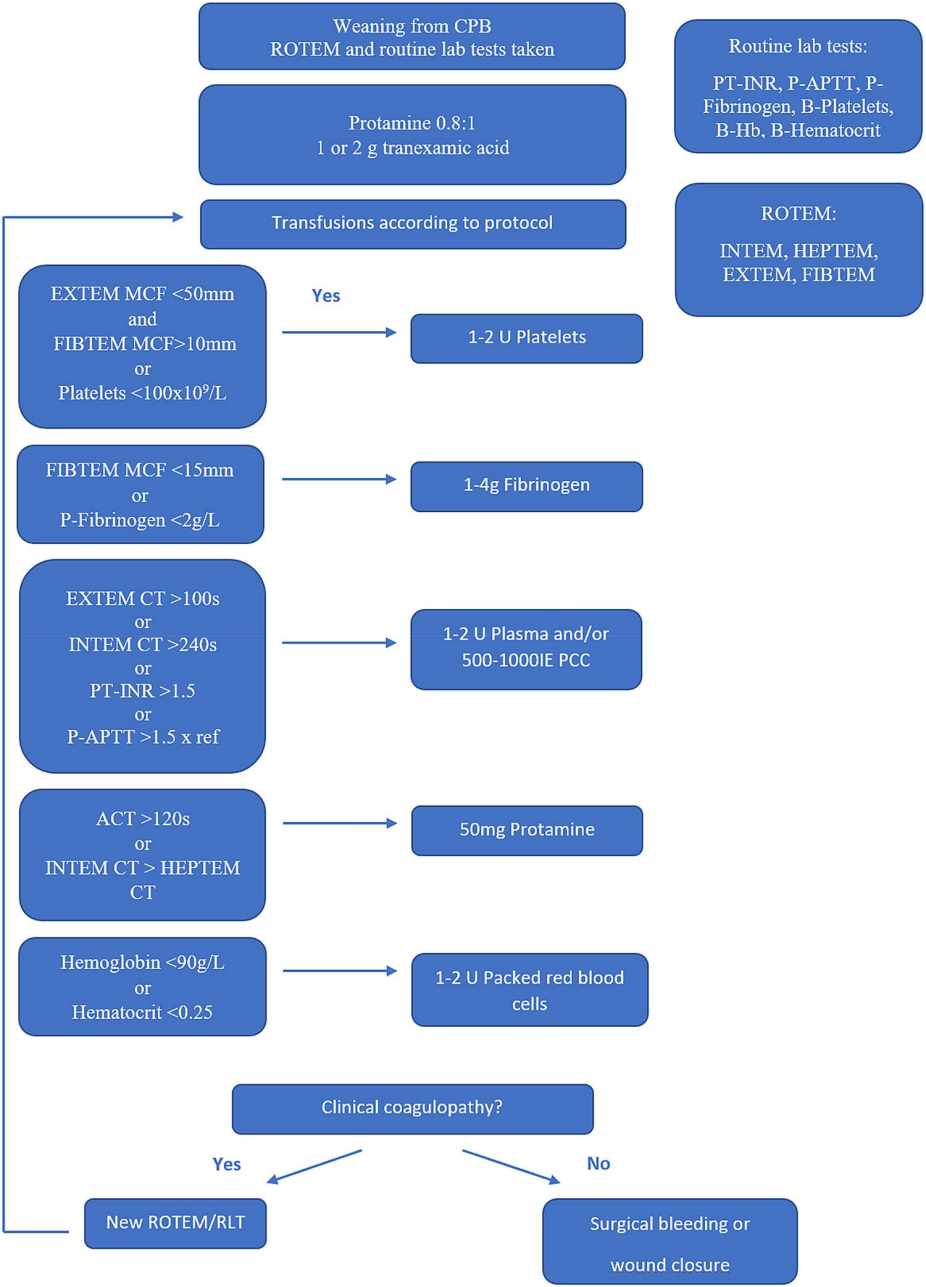
Transfusion protocol.

All patients with acute aortic syndromes (*e.g.* ATAAD and intramural hematomas) referred to our clinic during the study period were registered and screened for inclusion (Fig. 1). Routinely, a ROTEM Delta (Tem Innovations GmbH, Germany) and standard lab test guided bleeding management protocol was used at our clinic (Fig. 2). Red blood cell transfusions were given at B-Hemoglobin < 90 g/L. Platelets were administered at maximum clot firmness (MCF) EXTEM < 50 mm and MCF FIBTEM > 10 mm or platelet count < 100 × 10^9^/L. Fibrinogen and/or plasma were used at MCF FIBTEM < 15 mm or P-fibrinogen < 2 g/l. Plasma or prothrombin complex concentrate (PCC) were used at coagulation time (CT) EXTEM > 100 s, CT INTEM > 240 s, P-PT(INR) > 1.5 or P-APTT > 1.5 × normal value. Additional Tranexamic acid was used when maximum lysis (ML) exceeded 15%. However, final decision regarding transfusions was at the surgeon’s discretion.

### Endpoints

The primary endpoints were measurements of ROTEM values of clotting time (CT) in INTEM, EXTEM, and HEPTEM and maximum clot firmness (MCF) in EXTEM and FIBTEM. Secondary endpoints were need for transfusion with blood products, procoagulants used, 30-day mortality, bleeding at 24 h, and reoperation for bleeding.

### Definitions

An acute type A aortic dissection was defined as a separation of the aortic layers providing a true and a false lumen for the blood flow, presenting within 14 days of symptom onset and extending distally from the aortic root or ascending aorta (Stanford A). Surgical mortality was defined as death within 30 days of surgery. Intraoperative bleeding was defined as blood loss collected and quantified using intraoperative cell salvage and surgical gauze swabs. Postoperative stroke was defined as a permanent loss of neurological function caused by an ischemic event with or without confirmation with computed tomography or magnetic resonance imaging. Renal replacement therapy (RRT) was defined as any need for continuous hemofiltration or hemodialysis postoperatively. Cardiac tamponade was defined as pericardial effusion that required pericardial drainage or re-exploration.

### Biomarker measurements

Samples of blood were collected at six points in time: T_0_—anesthesia induction; T_1_—lowest core temperature (only collected in elective patients with core temperature ≤ 32 °C, n = 14); T_2_—just before protamine administration; T_3_- end of surgery; T_4_—24 h after surgery; T_5_—96–120 h after surgery. A central venous line was used for samples collected at T_0_, T_4_, and T_5_. A circuit from the cardiopulmonary bypass machine was used for samples collected at T_1_ and T_2_. A radial artery line was used for the samples collected at T_3_.

All samples were analyzed at the Dept. of Clinical Chemistry, Division of Laboratory Medicine, Skåne University Hospital, Lund, Sweden, on a ROTEM. Parameters tested were INTEM, EXTEM, HEPTEM, and FIBTEM and variables clotting time (CT), which is time from start until clotting is initiated in seconds (s) and maximum clot firmness (MCF), which is maximum diameter of clot in millimeters (mm).

### Surgical procedures

The surgical technique for ATAAD used at our center has been described previously^17^. Median sternotomy, cardiopulmonary bypass (CPB) with hypothermic circulatory arrest and cold blood cardioplegia was used in all cases. Heparin was reversed with protamine after weaning from CPB and transfusion of procoagulants and blood products initiated. The cooling strategy used in the elective controls were hemiarch procedure 25 °C; valve sparing root replacement (ad modum David): 30 °C; root replacement (ad modum Bentall) or aortic valve replacement with supracoronary replacement of the ascending aorta: 32 °C; isolated replacement of the ascending aorta: 32–36 °C.

During the study period, all patients received preoperative tranexamic acid (2–3 g preoperatively and 1–2 g after termination of CPB for a total of 4 g) to prevent hyperfibrinolysis. A Dideco Electa (Sorin Group, Electa, Italy) cell saver machine was used to process and re-infuse salvaged blood. Heparin dose was calculated using the Hepcon HMS Plus system (Medtronic, Minneapolis, MN, USA) to reach an activated clotting time (ACT) of > 480 s and monitored serially during CPB to maintain ACT > 480 s with additional heparin added if necessary. Antithrombin was administered if the heparin dose response slope was low (< 70 s/U/ml) and a subsequent test was conducted ten minutes later. Two patients in each group received 2000 IE of antithrombin during surgery. ACT was routinely controlled at the end of surgery, and additional protamine was given if ACT exceeded 120 s.

### Statistical analysis

Categorical variables were expressed as numbers and percentages. Continuous variables were expressed as mean (± SD) when normally distributed, otherwise as median [IQR]. ROTEM variables were visualized using box plots. ROTEM reference values are illustrated as dashed lines and shaded areas and are based on reference ranges by Lang et al.^20^. Proportions were compared using the chi-square test. Fisher’s exact test was used when the number of cases was less than 5, and continuous variables were evaluated using the Mann–Whitney test. Wilcoxon signed-rank test was used for analyzing related samples. Missing values were not analyzed. A *p*-value < 0.05 was considered statistically significant and analyses were performed using standard software (IBM Corp. Released 2019. IBM SPSS Statistics, Version 26. Armonk, NY: IBM Corp).

### Ethical approval

This study was approved by the Ethics Committee for Clinical Research at Lund University, Sweden (ref. 2015/197). Patient consent: Before inclusion, a consent was gathered from the patient or the next of kin (oral and/or written).

## Results

### Baseline and intraoperative data

Patient characteristics are presented in Table 1 and intraoperative data in Table 2. Bicuspid aortic valve was less common in the ATAAD group (0% vs 32%, *p* < 0.01), and proximal surgery was less extensive with fewer root replacements in the ATAAD group (*p* < 0.001) compared to the control group. Cardiopulmonary bypass time was shorter in the ATAAD group (160 (± 36) min vs 208 (± 60) min, *p* < 0.01) as well as aortic cross-clamping (75 (± 31) min vs 139 (± 44) min, *p* < 0.001). The lowest core temperature was significantly lower in the ATAAD group (20.0 (± 2.8)°C vs 30.5 (± 3.1)°C, *p* < 0.001).

**Table 1:** Preoperative characteristics.

	ATAAD (n = 23)	Control (n = 20)	p-value
Age (years)	64.6 ± 11.1	59.2 ± 14.2	0.17
Sex (Female)	7 (30%)	3 (15%)	0.29
Hypertension	14 (61%)	11 (55%)	0.70
Marfan	0 (0%)	2 (10%)	0.21
Other connective disorder	0 (%)	0 (0%)	1
Bicuspid valve	0 (0%)	6 (32%)	< 0.01
Aortic aneurysm	6 (26%)	20 (100%)	< 0.001
Diabetes	0 (0%)	1 (5%)	0.47
Smoking history	7 (30%)	4 (27%)	1
Chronic kidney disease	1 (4%)	0 (0%)	1
COPD	0 (0%)	1 (5%)	0.47
Stroke	1 (4%)	0 (0%)	1
Aspirin	3 (13%)	2 (10%)	1
Hyperlipidemia	1 (4%)	6 (30%)	0.04

Values are expressed as numbers (%) or mean ± SD.

*ATAAD* acute aortic dissection, *COPD* chronic obstructive pulmonary disease.

**Table 2:** Intraoperative characteristics.

	ATAAD (n = 23)	Control (n = 20)	p-value
**Proximal surgical technique**			< 0.001
Supracoronary graft	21 (91%)	3 (6%)	
Supracoronary graft + AVR	1 (4%)	1 (5%)	
Root replacement	1 (4%)	16 (80%)	
Valve sparing root replacement	0 (0%)	8 (40%)	
Bentall	1 (4%)	8 (40%)	
**Distal surgical technique**			0.04
Supracoronary graft	21 (91%)	16 (80%)	
Hemiarch	0 (0%)	4 (20%)	
Arch	2 (9%)	0 (0%)	
**Arterial cannulation**			< 0.001
Femoral	18 (78%)	0 (0%)	
Arch	1 (4%)	17 (85%)	
Other	4 (17%)	3 (15%)	
Symptom duration (h)	7.0 (3.9)		
Operation time (min)	298 ± 72	322 ± 79	0.10
CPB time (min)	160 ± 36	208 ± 60	< 0.01
Cx time (min)	75 ± 31	139 ± 44	< 0.001
HCA time (min)	21.6 ± 10.5	3.6 ± 8.2	< 0.001
**SCP modality**			< 0.001
Circulatory arrest	14 (61%)	0 (0%)	
SABP	9 (39%)	4 (20%)	
SCP time		0	< 0.001
Lowest core temp (°C)	20.0 ± 2.8	30.5 ± 3.1	< 0.001
Circulatory arrest time (min)	17.9 ± 5.8	0	< 0.01
Intraoperative bleeding (ml)	2407 [1805–3204]	1410 [917–1920]	< 0.001

Values are expressed as numbers (%) , mean ± SD or median [IQR].

*ATAAD* acute aortic dissection, *AVR* aortic valve replacement, *CPB* cardiopulmonary bypass, *Cx* crossclamp, *HCA* hypothermic circulatory arrest, *SCP* selective cerebral perfusion, *SABP* selective antegrade brain perfusion.

### Biomarker measurements

**Figure 3:**
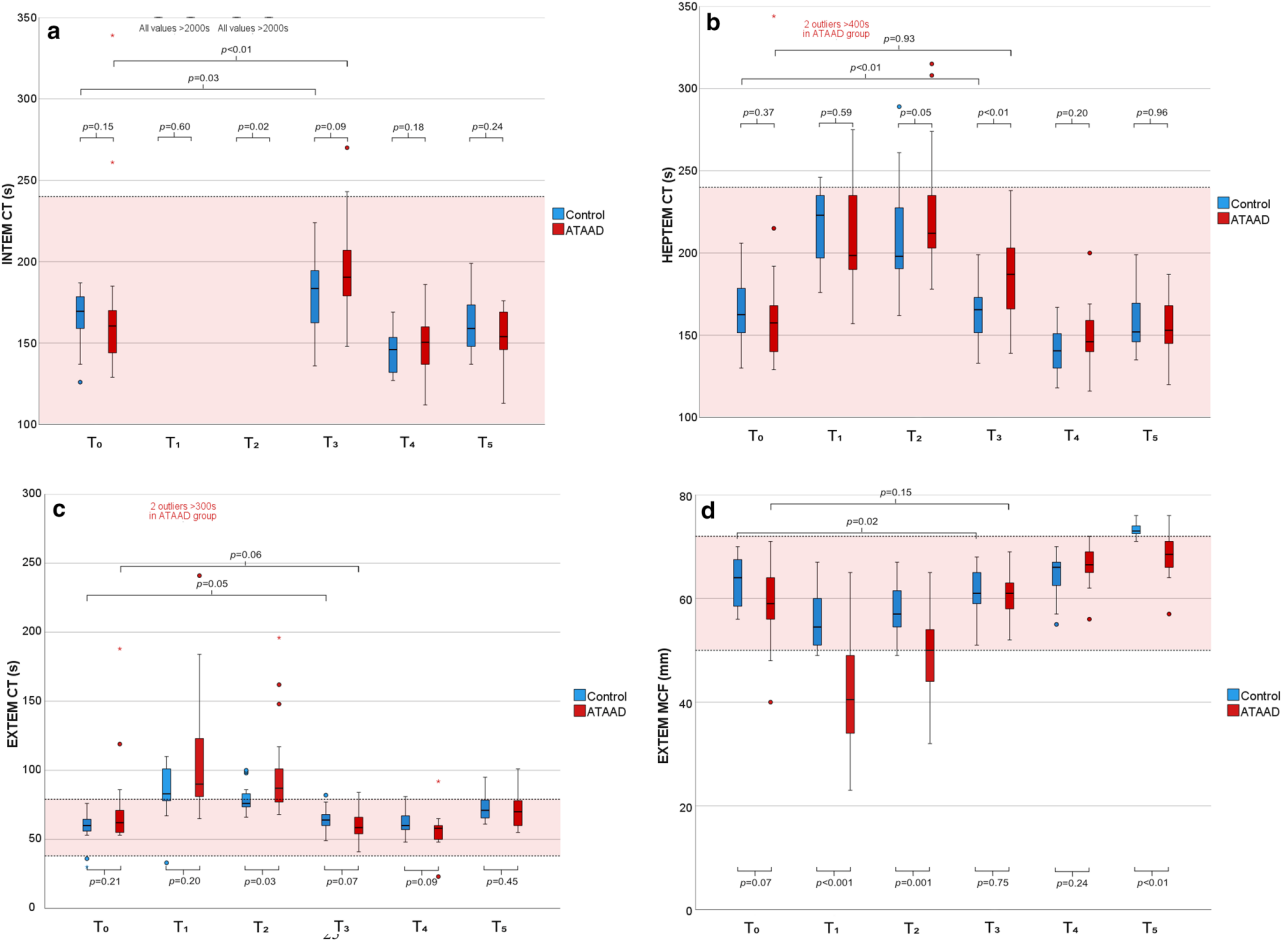

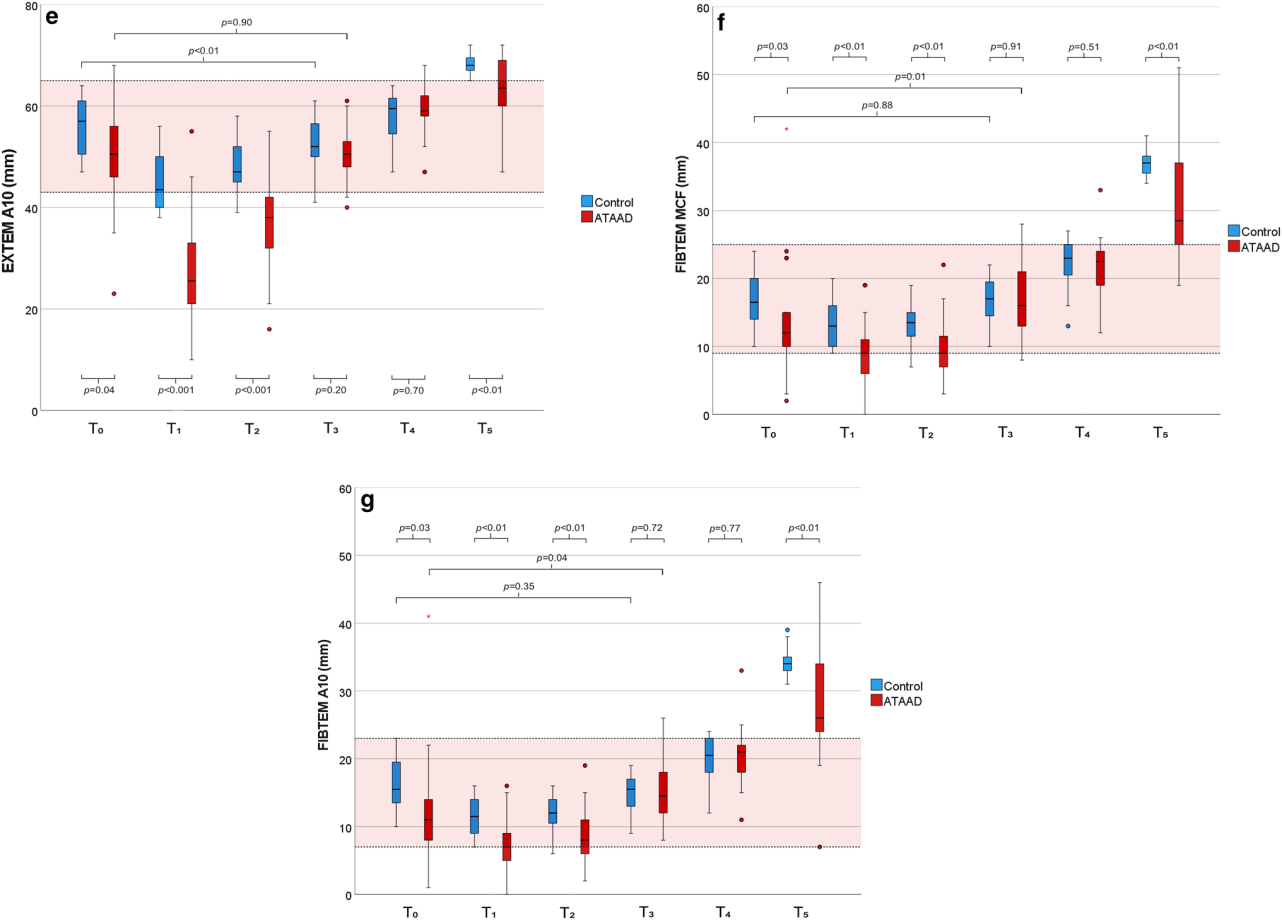
ROTEM analyses. (**a**) INTEM CT. (**b**) HEPTEM CT. (**c**) EXTEM CT. (**d**) EXTEM MCF. (**e**) EXTEM A10. (**f**) FIBTEM MCF. (**g**) FIBTEM A10.

ROTEM data is presented in Fig. 3a−g. At T_0_ there was no significant difference in clotting time (CT) in INTEM (Fig. 3a) or HEPTEM (Fig. 3b) between groups (159 s [140–170] vs 170 s [159–180], *p* = 0.08 and 156 s [138–168] vs 163 s [150–180], *p* = 0.21). During CPB, HEPTEM CT increased markedly in both groups and was significantly higher in the ATAAD group at T_2_ (212 s [202–239] vs 198 s [193–236], *p* = 0.05). At the end of surgery (T_3_), the HEPTEM CT recovered, although not to preoperative levels and was significantly higher in the ATAAD group (187 s [166–203] vs 166 s [151–173], *p* < 0.01). The CT in INTEM and HEPTEM was significantly more prolonged in the ATAAD group at T_3_ compared to T_0_ (191 s [179–211] vs 159 s [140–170], *p* < 0.01 and 187 s [166–203] vs 156 s [138–168], *p* < 0.01). In the control group, INTEM CT was significantly longer at T_3_ compared to T_0_ (184 s [162–196] vs 170 s [159–180], *p* = 0.03) but not HEPTEM CT (166 s [151–173] vs 163 s [150–180], *p* = 0.93).

#### EXTEM

There was a significant difference in EXTEM CT (Fig. 3c) at T_2_ (87 s [76–107] vs 76 s [73–84], *p* = 0.03) and a trend towards it at T_3_ (59 s [54–67] vs 64 s [60–68], *p* = 0.07). At the end of surgery, CT in the control group was significantly longer than preoperative levels (64 s [60–68] vs 60 s [56–65], *p* = 0.05) and in the ATAAD group there was a trend towards shorter CT compared to preoperative (59 s [54–67] vs 62 s [56–71], *p* = 0.06).

The maximum clot firmness (MCF) in EXTEM (Fig. 3d) was lower in the ATAAD group compared to the control group at T_1_, T_2,_ and T_5_ (41 mm [34–41] vs 55 mm [51–61], *p* < 0.001, 50 mm [44–55] vs 57 mm [54–62], *p* = 0.001 and 69 mm [66–72] vs 73 mm [72–74], *p* =  < 0.01, respectively) and had a similar trend at T_0_ (59 mm [56–65] vs 64 mm [58–68], *p* = 0.07). EXTEM MCF was also lower in the control group at the end of surgery compared to preoperative levels (61 mm [59–65] vs 64 mm [58–68], *p* = 0.02) but not in the ATAAD group (61 mm [58–64] vs 59 mm [56–65], *p* = 0.15). Clot firmness at 10 min (A10) showed similar pattern and is presented in Fig. 3e.

#### FIBTEM

The preoperative FIBTEM MCF (Fig. 3f) was significantly lower in the ATAAD group compared to the control group (12.0 mm [10.0–15.0] vs 16.5 mm [13.5–20.0], *p* = 0.03), and the pattern persisted during the operation (at T_1_ 9.0 mm [5.5–11.5] vs 13.0 mm [10.0–16.3], *p* < 0.01 and at T_2_ 9.0 mm [7.0–12.0] vs 13.5 mm [11.3–15.0], *p* < 0.01). There was no difference in FIBTEM MCF at the end of surgery (T_3_) or the first postoperative day (T_4_) (*p* = 0.91 and *p* = 0.51, respectively), however at T_5_, MCF was again lower in the ATAAD group (28.5 mm [25.0–37.3] vs 37.0 mm [35.0–38.0], *p* < 0.01). A10 clot firmness followed the same pattern and is shown in Fig. 3g.

### Bleeding, transfusions and medical management

Data presented in Table 3 reveal that patients in the ATAAD group received significantly more transfusions and procoagulants during surgery and the first 24 h after surgery. Transfusion of packed red blood cells (3.3U (± 4.0) vs 1.2U (± 1.5), *p* = 0.03), platelets (4.3U (± 1.7) vs 2.5U (± 1.6), *p* = 0.001) and FFP (2.4U (± 2.6) vs 0.6U (± 1.2), *p* < 0.01) was larger in the ATAAD group. The use of fibrinogen (6.0 g (± 3.4) vs 3.1 g (± 2.5), *p* < 0.01), PCC (1780 IU (± 1050) vs 775 IU (± 896), *p* < 0.01) and recombinant factor VIIa (5 (22%) vs 0 (0%) patients, *p* = 0.03) also were significantly larger in the ATAAD group. No patient was reoperated for bleeding in both groups and postoperative bleeding volumes at 12 h (492 ml (± 235) vs 498 ml (± 175), *p* = 0.64) and 24 h (810 ml (± 400) vs 740 ml (± 215), *p* = 0.78) did not show any difference between groups.

**Table 3:** Transfusion and postoperative characteristics.

	ATAAD (n = 23)	Control (n = 20)	p-value
Early mortality	1 (4%)	0 (0%)	1
**Chest output (ml)**			
12 h	492 ± 235	498 ± 175	0.64
24 h	810 ± 400	740 ± 215	0.78
**Transfusions**			
Packed red blood cells (U)	3.3 ± 4.0	1.2 ± 1.5	0.03
Intraoperative	1 [0–2]	0 [0–0]	0.003
Platelets (U)	4.3 ± 1.7	2.5 ± 1.6	0.001
Intraoperative	3.8 ± 1.2	1.8 ± 1.6	< 0.001
Fresh frozen plasma (U)	2.4 ± 2.6	0.6 ± 1.2	< 0.01
Intraoperative	0 [0–2]	0 [0–0]	0.70
Fibrinogen (g)	6.0 ± 3.4	3.1 ± 2.5	< 0.01
Intraoperative	5.8 ± 2.4	2.9 ± 2.3	< 0.001
Novoseven	5 (22%)	0 (0%)	0.03
Intraoperative	5 (22%)	0 (0%)	0.03
Antithrombin	2 (9%)	2 (10%)	1
Tranexamic acid (g)	3.5 ± 0.9	3.9 ± 0.4	0.03
Desmopressin (µg)	11.6 ± 14.1	15.8 ± 14.8	0.31
Intraoperative	11.6 ± 14.1	15.8 ± 14.8	0.31
PCC (IE)	1780 ± 1050	775 ± 896	< 0.01
Intraoperative	1583 ± 985	575 ± 815	< 0.001
Re-exploration for bleeding	0 (0%)	0 (0%)	1
Postoperative stroke	5 (22%)	1 (5%)	0.19
Dialysis (RRT)	3 (13%)	0 (0%)	0.24
Prolonged ventilation (> 48 h)	8 (35%)	0 (0%)	< 0.01
ICU stay (days)	6.7 ± 6.8	1.4 ± 0.8	< 0.001

Values are expressed as numbers (%) or mean ± SD.

*ATAAD* acute aortic dissection, *PCC* prothrombin complex concentrate, *RRT* continuous renal replacement therapy, *ICU* intensive care unit.

## Discussion

The results of this study demonstrate that there are predictable and quantifiable changes in ROTEM values during surgery in ATAAD and elective aortic surgery with CPB. Surgery significantly and negatively impacted all ROTEM values assessed in this study (EXTEM CT, INTEM CT, HEPTEM CT, EXTEM MCF, and FIBTEM MCF). The greatest impairment in coagulation parameters occurred consistently in patients with ATAAD. This study demonstrated that ATAAD caused an activation of the coagulation shown in ROTEM prior to surgery (T_0_), which developed to a coagulopathy during CPB (T_1_ and T_2_) and was not fully recovered compared to elective controls at wound closure (T_3_) despite significantly greater use of procoagulants and transfusions. Our ROTEM-guided transfusion protocol does not seem to catch the full need of transfusions as most of our patients received more transfusions than what ROTEM would suggest. However, bleeding volumes and the need for re-exploration for bleeding or tamponade did not differ and had favorable outcomes in both groups.

Aortic dissection leads to blood being exposed to tissue factor, extracellular collagen, and other subendothelial structures that activate the coagulation process. This is evident by the decreased MCF in FIBTEM indicating consumption of fibrinogen. MCF in EXTEM also was decreased but did not reach the level of significance. Both EXTEM and INTEM CT showed a trend towards longer clotting time indicating reduced amounts of clotting factors. Combined, this indicates an established activation of coagulation when ATAAD develops resulting in a consumption coagulopathy. During surgery, both groups showed similar trends in all ROTEM variables, but the ATAAD group had consistently more impaired values, which could indicate that the dissection consumed coagulation factors, platelets, and fibrinogen prompted by more profound hypothermia.

At the end of surgery, both groups showed similar ROTEM findings. The ATAAD group had longer HEPTEM CT than the control group, but not INTEM CT. This indicates a reduction of factors in the intrinsic pathway in the ATAAD group and a remaining heparin effect in the control group. In the ATAAD group, FIBTEM is normalized and equal to the control group. These findings are in line with similar work by Liu et al. and Guan et al. who did serial TEG analysis on patients with ATAAD^21,22^. Data in both studies demonstrated that ATAAD initiates a consumption coagulopathy, and surgery affects fibrinogen and clotting factors more than platelets. However, TEG is not able to detect differences between intrinsic and extrinsic pathways. The studies lacked either a control group or did not provide differences between samples with heparinase.

Postoperatively, the patient was hypercoagulable in both groups, primarily in terms of FIBTEM, which may be explained by an increase in fibrinogen levels caused by inflammation^23^. ROTEM at day 4–5 (T_5_) showed normal CT in INTEM and a prolonged CT in EXTEM in both groups. The MCF was elevated in FIBTEM and EXTEM. The increase is likely driven by high fibrinogen levels. The prolonged CT in EXTEM indicates a higher threshold for extrinsic pathway activation, likely induced by increased activity by inhibiting factors such as antithrombin, protein C, and protein S. This is supported by the normal PT-INR and increased antithrombin at day five as shown previously^17^.

Point-of-care testing with either ROTEM or tromboelastography (TEG) has been proven to reduce the need for red blood cell transfusion and reduce bleeding in cardiac surgery^10^. The clotting time (CT) of INTEM and EXTEM test the same pathways as activated partial thromboplastin time (APTT) and prothrombin time (PT-INR), respectively. Maximum clot firmness (MCF) in both INTEM and EXTEM is an estimate of platelet number and/or function combined with fibrinogen levels while MCF in FIBTEM is primarily a measurement of fibrinogen levels^11^. One of the main benefits of ROTEM are its fast results compared to compared to routine plasma-based laboratory coagulation tests (RLT) which enables serial testing of the coagulation and allows for faster response on changes in the coagulation during and after surgery, and our results generated by ROTEM are in line with previous reports using RLT^17,24^. However, one main difference between ROTEM and RLT is that RLT has well-established quality assurance programs with imprecision results, coefficient of variation (CV%) < 5%, which is more difficult to achieve for a whole-blood system like ROTEM, reflected in the reference values that are wider in ROTEM counterparts. Another benefit of RLT is that ROTEM is harder to interpret and in the setting of complex coagulopathy, the use of both RLT and ROTEM provides a more nuanced picture^25^.

To be able to use ROTEM as a substitute for RLT in a transfusion protocol it needs to adequately identify differences in coagulation seen in RLT. When comparing our ROTEM data with RLT in the same cohort, we find that ROTEM does not detect as many pathologies as RLT does. ROTEM identified all cases with low levels of fibrinogen, a finding supported by previous studies^13,26^. However, low levels of clotting factors and platelets seem to be underdiagnosed by ROTEM. In this study, ROTEM suggests that 35% of the patients require PCC or FFP, but RLT indicates that all patients have decreased levels of coagulation factors. This is also demonstrated by Rugeri et al.^26^ who showed poor correlation between CT and APTT/PT-INR. Platelet levels were also underdiagnosed by ROTEM, where only one out of seven thrombocytopenic patients were detected compared to RLT. This could be due to the fact that ROTEM is a functional test and do not specifically correspond to each step in the coagulation cascade.

Our ROTEM-guided transfusion protocol was introduced in 2015. It follows a similar structure to previously published protocols^9,10,12^. The adherence to protocol in this study, however, was not always optimal. When analyzing the transfusions, all patients in the ATAAD group received platelets, fibrinogen, and PCC and/or FFP. Compared to the transfusion protocol, all patients in the ATAAD group met the criteria for PCC/FFP substitution, > 80% for fibrinogen, but only 50% met the criteria for platelets and RBC at T_2_. This could be interpreted in different ways: either pre-emptive transfusions were used to prevent coagulopathy or the protocol was bypassed due to clinical coagulopathy not detected by the protocol. As mentioned earlier, ROTEM failed to identify the patients with low levels of coagulation factors or platelets indicating that the ROTEM-guided algorithm used in routine surgery may not be directly translated to ATAAD or other complex surgery. Also, our ROTEM does not contain platelet function analysis, which may explain why the algorithm was not followed for platelet transfusion.

Although ROTEM has been proven to reduce transfusions in cardiac surgery, some studies show no predictive value of ROTEM^11,14–16,27^. One potential reason might be its imprecision. In elective surgery, a preoperative test enables the patient to act as its own control, and changes in ROTEM could better be interpreted in that context. However, in the acute setting of surgery for ATAAD, the patients’ preoperative values are impaired, and preoperative levels have a limited value. This is supported by several studies of ROTEM and TEG showing that comparing pre- and post CPB levels is better at predicting bleeding rather than a post CPB ROTEM/TEG analyzed with predetermined cut-off values^11,14–16,27^.

There are several limitations to this study. First, the sample size is not large leaving room for type II errors. However, including more subjects would require a longer inclusion period, and smaller differences found in a larger cohort may have questionable clinical value. The use of a control group undergoing aortic surgery is of benefit, but a better-matched surgical procedure with deep hypothermic circulatory arrest could possibly have given even more insights. Adherence to the transfusion protocol was impaired, and the true effect of a ROTEM-guided protocol could not be evaluated. However, the aim of this study was originally not intended for evaluating the ROTEM-guided transfusion protocol, and the results provided are merely indicative, requiring further studies.

## Conclusion

ATAAD induces an activation of the coagulation system that affects all three subsystems: the extrinsic, the intrinsic and the common pathway. ROTEM does not adequately catch the full coagulopathy in ATAAD. A transfusion protocol in ATAAD should be specifically created to target this complex coagulopathic state and ROTEM does not negate the need for routine laboratory tests.

## Data Availability

The datasets generated and/or analysed during the current study are not publicly available due to limitations in the ethics approval but are available from the corresponding author on reasonable request.

## Abbreviations

ROTEM: Rotational thromboelastometry
INTEM: Intrinsic thromboelastometry
EXTEM: Extrinsic thromboelastometry
HEPTEM: Heparinase thromboelastometry
FIBTEM: Fibrinogen thromboelastometry
MCF: Maximum clot firmness
CT: Clotting time
ATAAD: Acute type A aortic dissection
INR: International normalized ratio
APTT: Activated plasmothrombin time
